# Management of a Severe L4/5 Andersson Lesion with Three-Column Instability in Ankylosing Spondylitis Using Single-Stage Circumferential Reconstruction: A Case Report

**DOI:** 10.3390/jcm15176713

**Published:** 2026-08-29

**Authors:** Chang-Sheng Liao, Xia Chen, Zhong-Jie Zhou

**Affiliations:** Department of Orthopaedics, West China Hospital, Sichuan University, Chengdu 610041, China; 15603441409@163.com (C.-S.L.); 13438502627@163.com (X.C.)

**Keywords:** ankylosing spondylitis, Andersson lesion, pseudarthrosis, circumferential reconstruction, three-column instability, lumbar spine

## Abstract

**Background/Objectives:** Andersson lesions are uncommon discovertebral complications of ankylosing spondylitis and may be overlooked when a new mechanical pain pattern is attributed to chronic inflammatory disease. This report describes the diagnostic reasoning, operative decision-making, and short-term outcome of a severe lumbar lesion with three-column instability. **Methods:** A 38-year-old woman with a 15-year history of ankylosing spondylitis presented with progressive low-back pain, left L5 radiculopathy, and marked functional decline without major trauma. Radiographs, computed tomography, and magnetic resonance imaging were used to characterize the lesion and assess spinal instability and neural compression. A Cawley type E Andersson lesion at L4/5 with severe three-column instability was diagnosed. The patient underwent single-stage posterior–anterior circumferential reconstruction consisting of L4/5 decompression, long-segment L2-S2 fixation, anterior debridement, and tricortical iliac crest grafting. **Results:** A postoperative gastrocnemius intramuscular venous thrombosis was detected and treated with anticoagulation. At 3 months, the activity-related visual analog scale score improved from 8 to 1, the Oswestry Disability Index improved from 68% to 18%, independent ambulation was restored, and imaging showed stable instrumentation with progressive osseous bridging. At the latest 9-month telephone follow-up, the patient reported maintained pain relief, mobility, and daily function; no new imaging was available. **Conclusions:** A change from inflammatory to mechanical pain or the development of neurologic findings in ankylosing spondylitis should prompt targeted imaging for an Andersson lesion. In carefully selected patients with severe three-column instability and substantial anterior column deficiency, circumferential reconstruction may provide effective short-term stabilization and functional recovery. Because radiographic follow-up was limited to 3 months, definitive fusion and long-term construct durability cannot be established.

## 1. Introduction

Andersson lesions are destructive discovertebral lesions that occur in ankylosed spinal segments. They are generally interpreted as stress fracture-related pseudarthroses arising from the long-lever-arm biomechanics of a rigid spine, although inflammatory and degenerative mechanisms may also contribute [1,2,3]. Because the presenting symptom is often worsening back pain, diagnosis may be delayed when a new mechanical pain pattern is attributed to the underlying inflammatory disease. Computed tomography (CT) is particularly useful for defining fractures, osseous destruction, and pseudarthrosis, whereas magnetic resonance imaging (MRI) helps assess neural compression, marrow changes, and major differential diagnoses [1,3,4,5].

Treatment depends on lesion location, deformity, neurologic compromise, bone quality, and mechanical instability. Nonoperative management may be considered in selected stable lesions; however, displaced or unstable three-column lesions, progressive pain, neurologic deficits, or pseudarthrosis usually prompt surgical stabilization. Reported strategies include posterior-only fixation, circumferential reconstruction, and minimally invasive combined approaches, but the available evidence is dominated by retrospective series and case reports [6,7,8,9,10,11,12].

We describe a type E L4/5 Andersson lesion in a relatively young woman with long-standing ankylosing spondylitis, osteoporosis, severe three-column instability, and left L5 radiculopathy. The report focuses on diagnostic reasoning and the rationale for single-stage posterior–anterior circumferential reconstruction; the postoperative gastrocnemius intramuscular venous thrombosis is reported as an important perioperative complication. This report was prepared in accordance with the CARE framework for case reports.

## 2. Case Presentation

### 2.1. Patient Information and Clinical Findings

A 38-year-old woman with a 15-year history of ankylosing spondylitis was admitted in March 2024 because of low back pain lasting more than 3 years, with substantial worsening during the preceding year. The pain had become mechanical, was aggravated by activity, and was accompanied by radiating pain and numbness in the left lower limb. She reported pain scores of 4/10 at rest and 8/10 during activity. Walking tolerance had progressively decreased, and she was wheelchair-dependent at presentation. She denied any major preceding spinal trauma.

She had received pharmacological treatment for ankylosing spondylitis throughout the disease course. Celecoxib (Celebrex; Pfizer Pharmaceuticals LLC, Vega Baja, PR, USA) and diclofenac sodium (Voltaren; Beijing Novartis Pharma Co., Ltd., Beijing, China) were used as needed for symptomatic relief. She had previously received sulfasalazine (Shanghai Sine Tianping Pharmaceutical Co., Ltd., Shanghai, China) at a maintenance dose of 2.0 g/day (1.0 g twice daily) for approximately 5 years and had subsequently been treated with adalimumab (Qletli; Bio-Thera Solutions, Ltd., Guangzhou, China) at 40 mg subcutaneously every 2 weeks for the preceding 5 years. She had no history of long-term systemic glucocorticoid therapy. She had no history of previous spinal surgery or major medical comorbidity. Physical examination showed marked restriction of thoracolumbar and sacral motion and tenderness around both sacroiliac joints. Sensation was reduced over the lateral left lower leg and dorsum of the foot, the left extensor hallucis longus had Medical Research Council grade IV strength, and the left straight-leg-raising test was positive at 40 degrees. No pathologic reflexes were elicited. These findings were compatible with left L5 radiculopathy.

At admission, C-reactive protein and erythrocyte sedimentation rate values were 3.2 mg/L and 9 mm/h, respectively. HLA-B27 was positive. Routine blood counts, liver and kidney function tests, and baseline coagulation studies were within the local reference ranges. Dual-energy X-ray absorptiometry demonstrated osteoporosis (T-score, −2.7) (Table 1).

### 2.2. Diagnostic Assessment

**Figure 1:**
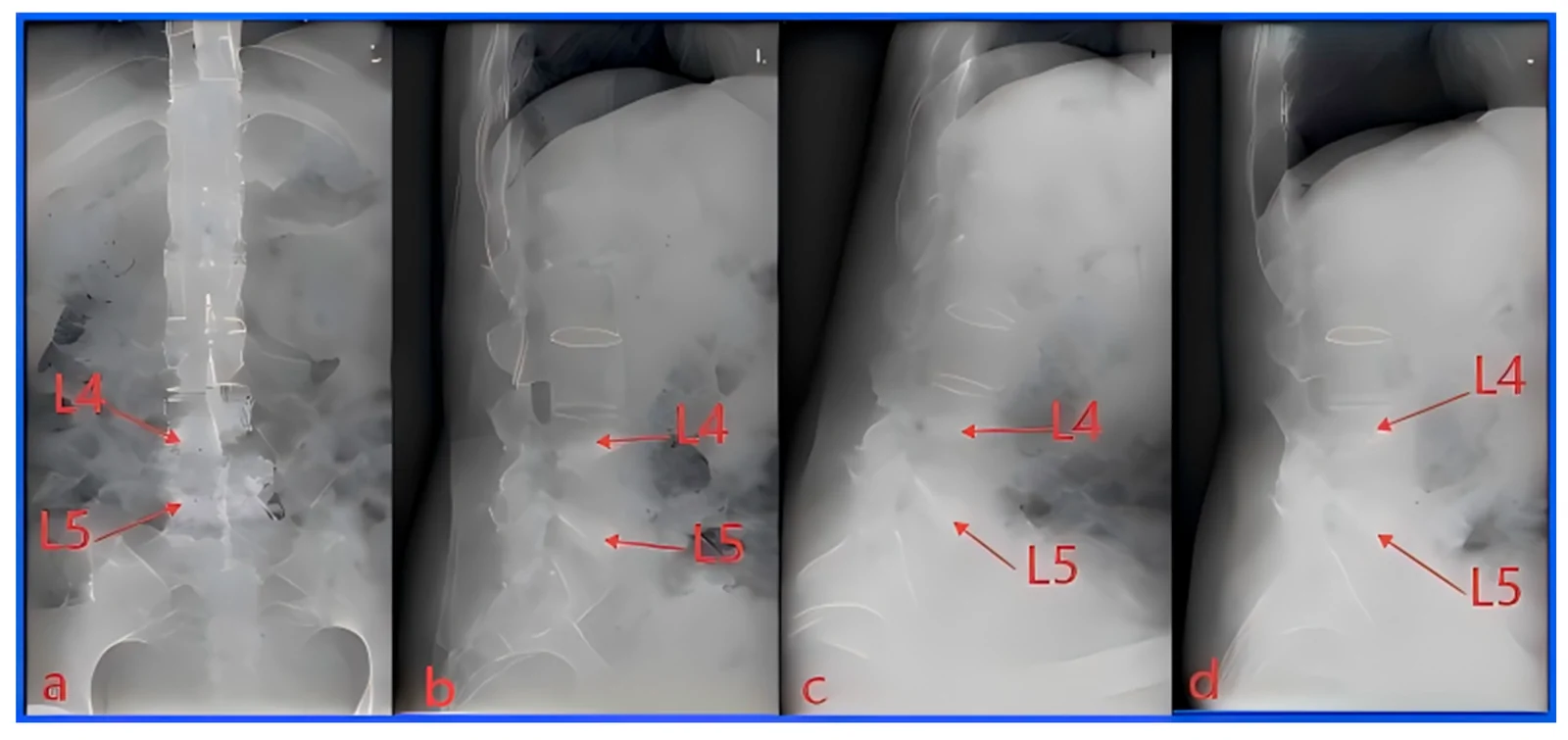
Preoperative lumbar radiographs. (**a**) Standing anteroposterior radiograph demonstrates the ankylosed “bamboo spine” and the abnormal transverse lesion at the L4/5 level. (**b**) Standing lateral radiograph shows the L4/5 lesion with anterior column involvement. (**c**) Flexion radiograph demonstrates no appreciable motion at the L4/5 segment. (**d**) Extension radiograph likewise shows no appreciable motion at the affected segment despite the structural disruption subsequently demonstrated on computed tomography. Red arrows indicate the L4 and L5 vertebral levels.

Standing anteroposterior and lateral radiographs showed a bamboo spine and a transverse radiolucent lesion at L4/5 with an anterior vertebral defect (Figure 1). Flexion-extension radiographs did not show appreciable motion; nevertheless, CT demonstrated structural disruption and pseudarthrosis in the ankylosed segment.

**Figure 2:**
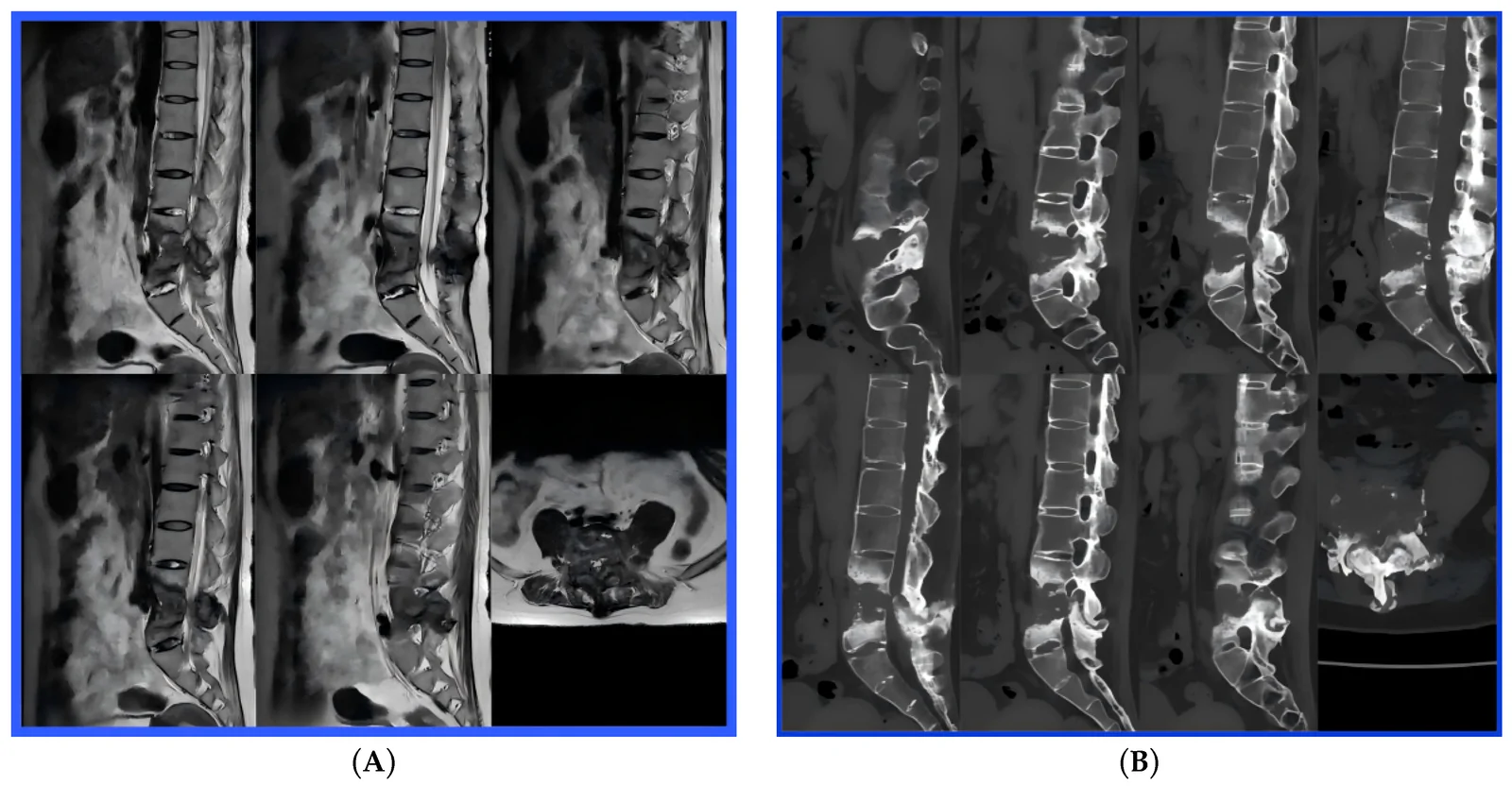
Preoperative advanced imaging. (**A**) Computed tomography demonstrates bilateral L4/5 pedicle fractures, anterior column bone destruction/resorption, and pseudarthrosis. (**B**) Magnetic resonance imaging demonstrates adjacent vertebral marrow edema and compression of the left L5 nerve root.

CT provided a detailed osseous assessment and showed bilateral L4/5 pedicle fractures, anterior column bone resorption, pseudarthrosis, and foraminal narrowing. MRI demonstrated loss of the normal L4/5 disk signal, adjacent vertebral marrow edema, and compression of the left L5 nerve root (Figure 2).

The principal differential diagnoses included active inflammatory disease, degenerative disk disease, infectious spondylodiscitis, and neoplastic destruction. The absence of systemic infectious features, non-elevated inflammatory markers, and the characteristic fracture–pseudarthrosis pattern favored a noninfectious Andersson lesion. Intraoperative specimens obtained during debridement were submitted for histopathological examination and microbiological tissue culture; both were negative for evidence of infection. Taken together, the clinical, laboratory, imaging, and intraoperative findings supported the final diagnosis of ankylosing spondylitis complicated by a Cawley type E L4/5 Andersson lesion with severe three-column instability and left L5 radiculopathy [2].

### 2.3. Therapeutic Intervention

NSAIDs were withheld 7 days before surgery. Baseline lower-extremity ultrasonography showed no pre-existing thrombosis. Because of immobility, ankylosing spondylitis, and the anticipated duration of major spine surgery, perioperative thromboprophylaxis was instituted according to the treating team’s protocol, including mechanical measures and prophylactic low-molecular-weight heparin. The documented osteoporosis was incorporated into the fixation strategy and postoperative bone health management.

A single-stage posterior–anterior circumferential reconstruction was performed under general anesthesia. The posterior stage was undertaken first. Total L4/5 laminectomy and left foraminotomy decompressed the spinal canal and left L5 nerve root. Long-segment pedicle-screw fixation from L2 to S2 was then used to distribute stress across the rigid and osteoporotic spine. Autograft and allograft were placed over the interlaminar and facet regions for posterior fusion.

**Figure 3:**
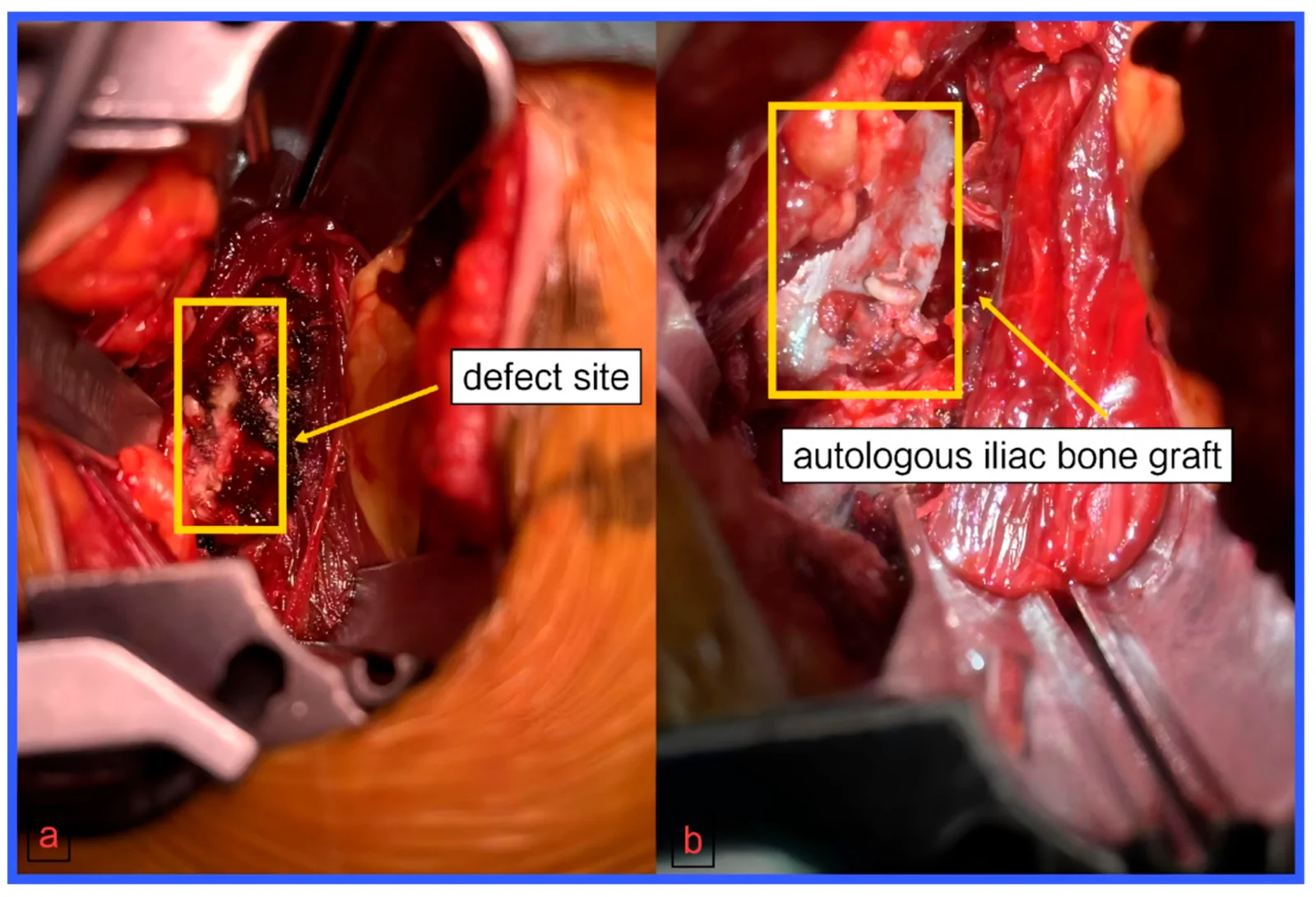
Intraoperative anterior reconstruction. (**a**) Anterior column defect after debridement of the L4/5 pseudarthrosis. (**b**) A shaped tricortical autologous iliac crest graft is placed to reconstruct the anterior column and provide structural support.

The patient was repositioned for a left extraperitoneal anterior approach. Fibrous tissue and inflammatory granulation within the pseudarthrosis were debrided, and specimens were submitted for histopathological examination and microbiological tissue culture; both were negative for evidence of infection. The endplates were then prepared, and a tricortical autologous iliac crest graft was shaped and implanted to reconstruct the anterior column (Figure 3). The operation lasted 410 min, the estimated blood loss was 1200 mL, and no transfusion was required.

### 2.4. Postoperative Course and Follow-Up

On postoperative day 1, left calf swelling prompted vascular ultrasonography, which demonstrated thrombosis in the gastrocnemius intramuscular veins. Therapeutic enoxaparin (1 mg/kg every 12 h) was started after multidisciplinary assessment of postoperative bleeding risk and was converted to oral rivaroxaban after 10 days. Anticoagulation was planned for at least 3 months or until full mobilization. Graduated compression stockings, limb elevation, analgesia, and structured early mobilization were used in parallel. Postoperative osteoporosis management consisted of oral calcium carbonate providing 600 mg of elemental calcium once daily with meals, vitamin D3 800 IU once daily, and teriparatide 20 μg administered subcutaneously once daily. Teriparatide was planned for 12 months, with continued calcium and vitamin D3 supplementation. The available retrospective record did not preserve exact day-by-day rehabilitation milestones; therefore, the precise timing of progression from assisted to independent ambulation could not be reliably reconstructed. Independent ambulation was confirmed at the 3-month clinical review. D-dimer subsequently decreased to 0.3 mg/L. No pulmonary embolism, wound complication, surgical-site infection, or neurologic deterioration were observed.

**Figure 4:**
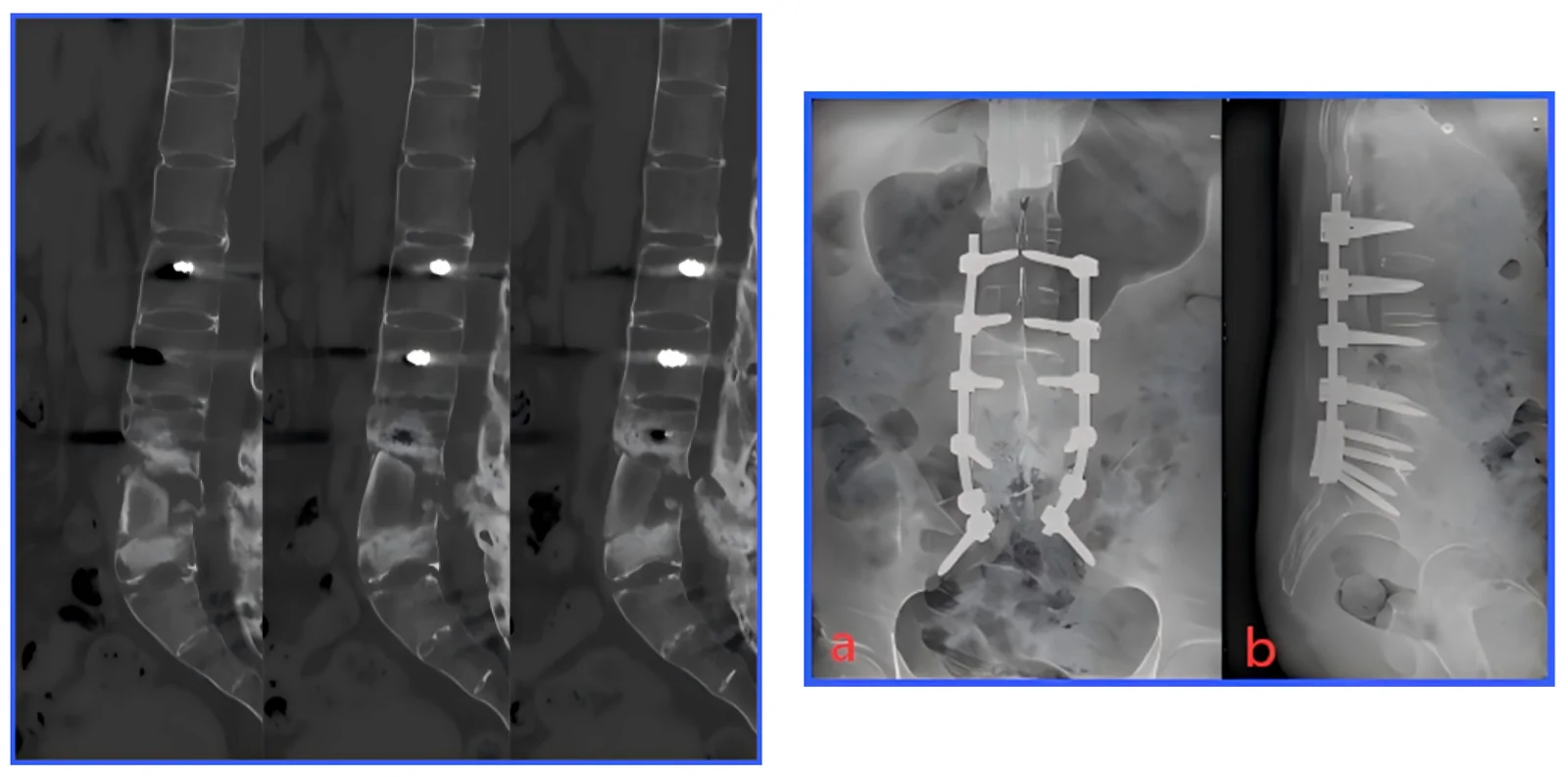
Early postoperative imaging. (**a**) Anteroposterior and lateral radiographs show maintained alignment and satisfactory long-segment instrumentation. (**b**) Computed tomography confirms appropriate position of the posterior fixation construct and anterior structural graft.

Early postoperative radiographs and CT showed maintained alignment, appropriate graft position, and stable instrumentation (Figure 4).

**Figure 5:**
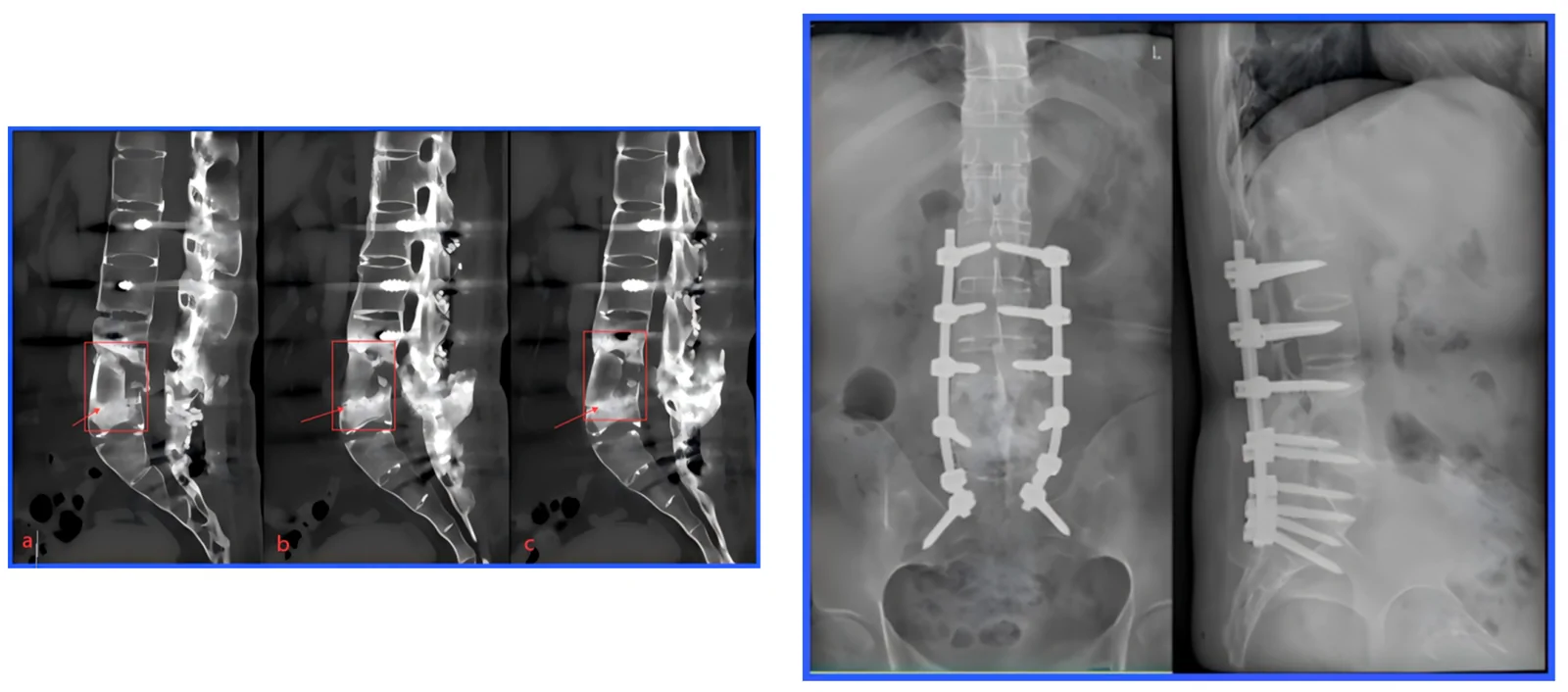
Imaging at the 3-month follow-up. (**a**) Anteroposterior radiograph shows maintained alignment and stable instrumentation without evident implant failure. (**b**) Lateral radiograph shows maintained alignment and stable instrumentation without evident implant failure. (**c**) Computed tomography demonstrates progressive trabecular–osseous bridging across the grafted L4/5 segment, consistent with early fusion progression rather than definitive mature fusion. Red boxes indicate the grafted L4/5 anterior column region, and red arrows indicate progressive trabecular–osseous bridging between the structural graft and the adjacent vertebral bodies.

At 3 months, activity-related low-back pain improved from a visual analog scale score of 8 to 1, and the Oswestry Disability Index improved from 68% to 18%. The patient regained independent ambulation for more than 1000 m and returned to light work. Radiographs showed maintained alignment without screw loosening, rod breakage, or loss of correction. CT demonstrated progressive trabecular bridging between the graft and adjacent vertebrae, consistent with early fusion progression rather than definitive long-term fusion (Figure 5). At the latest 9-month telephone follow-up, the patient reported sustained pain relief, independent ambulation, and satisfactory functional recovery. Because the 9-month assessment was conducted remotely, no contemporaneous physical examination or imaging was available; the latest radiographic evaluation remained the 3-month study.

### 2.5. Patient Perspective

The patient reported substantial improvement in pain, mobility, and the ability to perform daily activities. She expressed satisfaction with regaining independent walking and returning to light work. During the 9-month telephone follow-up, she continued to describe a satisfactory recovery and maintained daily function. Her subjective assessment was consistent with the measured improvements at 3 months, although no objective physical examination was performed at 9 months.

## 3. Discussion

### 3.1. Diagnostic Considerations

This case demonstrates a common diagnostic problem in ankylosing spondylitis: a new mechanical pain pattern and neurologic symptoms may be obscured by chronic inflammatory back pain. Andersson lesions are frequently associated with fracture through a fused segment, reactive sclerosis or erosions, and pseudarthrosis. The rigid spine behaves biomechanically like a long bone, and osteoporosis further reduces resistance to repetitive loading [1,3,13]. In the present patient, the absence of major trauma supports a fatigue-fracture mechanism.

Radiographs established the background of an ankylosed spine and suggested the transverse lesion, but CT and MRI were complementary. CT best demonstrated the bilateral pedicle fractures, anterior column defect, and pseudarthrosis; MRI defined the left L5 nerve root compression and marrow response. Infectious spondylodiscitis remained an important differential diagnosis because destructive discovertebral lesions in ankylosing spondylitis can mimic infection. In this patient, normal inflammatory markers, the absence of systemic infectious manifestations, the characteristic fracture–pseudarthrosis morphology, and negative intraoperative histopathological examination and tissue culture collectively supported a noninfectious Andersson lesion [4,5]. Nevertheless, negative microbiological and histopathological findings cannot absolutely exclude every low-grade infection, and the diagnosis should therefore be interpreted in the context of the complete clinical and radiographic picture.

### 3.2. Surgical Strategy

The goals of surgery are neural decompression, restoration of mechanical stability, and achievement of a durable fusion. A posterior-only approach can provide effective stabilization in many patients and may reduce approach-related morbidity [6,9,10]. However, substantial anterior column destruction, a large pseudarthrotic gap, severe three-column instability, and poor bone quality may justify anterior reconstruction in addition to long posterior fixation [6,7,8].

In this patient, posterior decompression directly addressed L5 radiculopathy, while fixation from L2 to S2 distributed load across multiple levels in an osteoporotic ankylosed spine. The anterior stage allowed direct debridement and structural grafting of the deficient anterior column. The combined procedure therefore addressed both neural and mechanical pathology during one anesthetic episode. Nevertheless, the case cannot demonstrate superiority over posterior-only or minimally invasive strategies; the approach should be individualized, and less invasive options may be appropriate when adequate debridement, reduction, fixation, and anterior support can be achieved [6,11,12].

### 3.3. Case-Based Context Within the Published Literature

Published reports illustrate that surgical management of Andersson lesions is heterogeneous and is largely driven by lesion morphology and mechanical demands rather than by a single universally accepted construct. Earlier work emphasized direct anterior debridement and fusion for discovertebral pseudarthrosis [7], whereas subsequent series demonstrated that posterior-only long-segment fixation can provide effective stabilization in appropriately selected lesions [8,10,11]. Circumferential strategies, including all-posterior circumferential fusion, have been described when direct management of a major anterior column defect is considered necessary [9]. More recent reports have also explored minimally invasive combined strategies to reduce approach-related morbidity [12]. A systematic review likewise found that the evidence base consists predominantly of retrospective series and case reports and does not establish the superiority of one approach for all patients [6]. Against this background, the present case is best viewed as an example of individualized reconstruction for the specific combination of severe three-column instability, a substantial anterior column defect, osteoporosis, and symptomatic L5 nerve root compression, rather than as evidence that circumferential reconstruction should be used routinely.

### 3.4. Perioperative Considerations and Bone Health

Venous thromboembolism risk should be considered in immobilized patients undergoing prolonged complex spine surgery, particularly in the setting of ankylosing spondylitis [14,15]. In this patient, baseline ultrasonography excluded pre-existing thrombosis, but symptomatic gastrocnemius intramuscular venous thrombosis was detected on postoperative day 1. Prompt ultrasonographic assessment and individualized anticoagulation were followed by an uncomplicated in-hospital course. This event is reported as a perioperative complication and should not be interpreted as evidence for a specific anticoagulation protocol.

Osteoporosis is also clinically relevant in ankylosing spondylitis because reduced bone strength coexists with a rigid spinal column and may increase fracture, fixation-failure, and nonunion risks [13]. The patient’s T-score of −2.7 influenced the choice of long-segment fixation and circumferential anterior support. Postoperatively, calcium carbonate (600 mg elemental calcium once daily), vitamin D3 (800 IU once daily), and teriparatide (20 μg subcutaneously once daily, planned for 12 months) were used as the bone health regimen. In this single case, the regimen should be interpreted as individualized osteoporosis management intended to support bone health during fusion rather than as evidence that a specific pharmacologic strategy improves fusion outcomes in Andersson lesions.

### 3.5. Limitations

This report has several limitations. First, it concerns one patient and cannot establish comparative effectiveness or causality. Second, objective radiographic follow-up was limited to 3 months. Although clinical status was updated at 9 months by telephone, no contemporaneous neurologic examination or imaging was available; mature fusion, long-term implant survival, and adjacent-segment complications therefore cannot be assessed. Third, bone-turnover markers and quantitative longitudinal imaging were not systematically collected. Fourth, although intraoperative histopathological examination and microbiological tissue culture were negative for evidence of infection, these studies cannot completely exclude every low-grade infectious process. Finally, the prophylactic low-molecular-weight heparin and subsequent rivaroxaban regimens reflect the local clinical context and should not be generalized as a universal protocol.

## 4. Conclusions

A change from inflammatory to mechanical pain, progressive disability, or new neurologic findings in ankylosing spondylitis should prompt evaluation for an Andersson lesion. In this patient with a type E L4/5 lesion, osteoporosis, left L5 radiculopathy, severe three-column instability, and major anterior column deficiency, single-stage posterior–anterior circumferential reconstruction was associated with marked short-term pain and functional improvement, stable instrumentation, and early trabecular bridging. This case supports circumferential reconstruction as an individualized option for selected mechanically severe lesions; long-term fusion and construct durability remain undetermined.

## Figures and Tables

**Table 1:** Clinical timeline.

Time Point	Clinical Course	Key Findings or Management
15 years before admission	Ankylosing spondylitis diagnosed	NSAIDs used as needed; sulfasalazine 2.0 g/day (1.0 g twice daily) for approximately 5 years; subsequent adalimumab 40 mg subcutaneously every 2 weeks during the preceding 5 years
>3 years before admission	Low-back pain developed	Gradual limitation of mobility
1 year before admission	Pain worsened and left-leg symptoms appeared	Progressive decline in walking tolerance; wheelchair dependence
March 2024	Hospital admission and diagnostic work-up	Left L5 radiculopathy; osteoporosis; L4/5 fracture–pseudarthrosis pattern on radiographs, CT, and MRI
Perioperative period	Single-stage circumferential reconstruction	Posterior decompression and L2-S2 fixation followed by anterior debridement and tricortical iliac crest grafting
Postoperative day 1	Left calf swelling	Gastrocnemius intramuscular venous thrombosis detected; therapeutic anticoagulation initiated
3 months	Clinical and radiographic review	VAS 8 to 1; ODI 68% to 18%; independent walking >1000 m; stable instrumentation and progressive osseous bridging
9 months (telephone follow-up)	Latest patient-reported follow-up	Pain relief and satisfactory functional recovery maintained; no new imaging available

## Data Availability

All data relevant to this case are contained within the article. Additional patient-level data are not publicly available because of privacy and confidentiality considerations.
